# Meniscal Allograft Transplants in Skeletally Immature Patients: A Systematic Review of Indications and Outcomes

**DOI:** 10.3390/healthcare11091312

**Published:** 2023-05-03

**Authors:** Marco Turati, Linda Boerci, Massimiliano Piatti, Laura Russo, Luca Rigamonti, Francesco Buonanotte, Aurelien Courvoisier, Giovanni Zatti, Daniele Piscitelli, Marco Bigoni

**Affiliations:** 1 School of Medicine and Surgery, University of Milano Bicocca, 20126 Milano, Italy; marco.turati@unimib.it (M.T.);; 2 Orthopedic Department, Fondazione IRCCS San Gerardo dei Tintori, 20900 Monza, Italy; 3 Transalpine Center of Pediatric Sports Medicine and Surgery, University of Milano-Bicocca, 20900 Monza, Italy; 4 Department of Paediatric Orthopedic Surgery, Hopital Couple Enfants, Grenoble Alpes University, 38700 Grenoble, France; 5 Department of Orthopaedic and Trauma, Policlinico San Pietro Hospital, 24036 Ponte San Pietro, Italy; 6 Department of Biotechnology and Biosciences, University of Milano Bicocca, 20126 Milano, Italy; 7 Department of Orthopedic Surgery, Mayo Clinic, Rochester, MN 55902, USA; 8 Physical Therapy Program, Department of Kinesiology, University of Connecticut, Storrs, CT 06269, USA

**Keywords:** anterior cruciate ligament, pediatric, physis status, skeletally immature, ACL reconstruction, ACLR, knee arthroscopy, pediatric sports medicine

## Abstract

Meniscal lesions in skeletally immature patients can lead to joint degradation and knee instability. Meniscal allograft transplant (MAT) surgery is a solution to maintain knee stability. There is a lack of consensus on MAT surgery outcomes in pediatric patients. A systematic review was conducted according to the PRISMA guidelines. PubMed, Scopus and EMBASE databases were searched from 1965 to June 2022. Studies were evaluated using the Newcastle–Ottawa Scale (NOS). Three studies were selected, and 58 patients were included (mean age 15.9 years) in total. The lateral meniscus was involved in 82.8% of all MAT surgeries. Post-meniscectomy syndrome and discoid meniscus were the main indications for MAT surgery. All studies reported improved subjective clinical scores and levels of sport after the surgery. The complication rate was 27.5%. Partial meniscectomy, meniscus knot removal, chondral defect treatment and lysis of adhesions were the most frequent procedures performed during reoperation. MAT surgery can improve clinical outcomes in pediatric patients with strictly selected indications. MAT surgery is safe when there are no limb asymmetries or malalignments, but it remains a challenging procedure with a high complication rate. Long-term follow-up is needed for definitive statements on the use of MAT in skeletally immature patients.

## 1. Introduction

The incidence of pediatric meniscal tears is rising due to increased participation in elite competition, early sports specialization and an increased ability to diagnose injuries [1]. In children, a substantial meniscal loss is more concerning than in adults because they require a functional knee for a longer lifespan, and early degenerative articular changes should be limited [2]. Currently, there is an international consensus on managing meniscal tissue lesions [3]. Meniscus allograft transplantation (MAT) is a recently developed treatment for patients who underwent large meniscectomy; it provides functional improvement and less pain of the knee joint. MAT increases the contact area of the joints and reduces peak contact stress [4].

The first true MAT was performed in 1972 [5]. Since then, this technique has gained popularity, and graft preservation and preparation methods have been developed. There is an embedded consensus about the typical individual for MAT: a young, active, non-obese subject who has stable knee ligaments, normal anatomic alignment and complains of a painful knee with meniscal deficiency [6]. Inflammatory arthritis, advanced joint arthritis, ligamentous instability, axial malalignment, smoking and obesity have been considered contraindications [7]. Some of these comorbidities can often be treated with simultaneous ligament reconstruction, osteotomy or cartilage restoration procedures. Skeletally immaturity is a relative contraindication for MAT. Some authors argue that physeal status should be assessed carefully because of the risk of physeal damage with consequent limb asymmetries and alignment deformities [8,9,10,11]. However, restoring knee anatomy may be an important factor in pain and OA progression. 

Most studies analyze patients between the age of 30 and 50 years, and the literature regarding the application of MAT in the pediatric population is lacking [12]. Due to the increasing number of children participating in sports, including elite sports, sports-related injuries in children and adolescents are becoming a more relevant issue. This may lead to a higher number of children with premature meniscus loss and consequent early progressive degenerative joint disease [7]. The discoid lateral meniscus (DLM) should also be considered: its prevalence ranges from 0.4% to 17%, with even higher percentages in Asian populations. The traditional treatment for a torn DLM was a total meniscectomy; midterm results were favorable, but long-term degenerative changes in the joint were shown [4]. This condition represents another possible cause of meniscus-deficient knees in youngsters, with further candidates for MAT. Due to the increased healing capacity of children, pediatric cohorts could potentially have better outcomes than adults following MAT, as suggested for meniscal repair [12]. Assuming the success of meniscal transplantation for adults is the same for immature patients is difficult, as the pediatric knee is still growing, and the results of the allograft in a growing joint are not well known. Improving data about the outcomes of such procedures could be helpful for surgeons during the decision-making process for the care of young patients.

Most studies regarding MAT consisted of retrospective series on adult patients, with a lack of high-level studies. In this context, no systematic review of the role of MAT in the pediatric and adolescent population was performed. We should consider that skeletally immature patients are different from adults, and there could be specific indications and surgical techniques for this population. Subsequently, as MAT is being more widely and frequently used, there is a need to assess its role in pediatric and adolescent patients. 

This study aims to perform a systematic review of MAT in skeletally immature patients to better understand which patients can benefit most from this surgery and to describe postoperative clinical outcomes regarding knee pain, function and the need for reoperation.

## 2. Materials and Methods

### 2.1. Research Question

Specific research questions were developed based on the Preferred Reporting Items for Systematic Review and Meta-analysis (PRISMA) guidelines [13]. The focused questions were “Are there some stated indications for meniscal allograft transplantation in young patients?” and ‘‘What are the clinical outcomes in terms of knee pain, function, and need for reintervention of meniscus allograft transplantation in these patients?”. A PICO was formulated as follows. Population: children and adolescents with meniscal injury; Intervention and Comparison: meniscal allograft transplantation surgery; Outcomes: pain, knee function, reinjury rate and need for reintervention.

### 2.2. Eligibility Criteria and Search Strategy

Original, full-text published studies in English were considered for inclusion if they investigated meniscal allograft transplantation and reported clinical outcomes in children and adolescents (<18 years old). No peer-reviewed articles, letters or editorials were excluded.

A literature search was conducted from 1965 to April 2023 in the PubMed/Medline (National Library of Medicine, Washington, DC, USA), Scopus and EMBASE databases using the following combination of keywords; (i) “meniscal” AND “transplant” AND “children”; (ii) “meniscal transplantation” AND “adolescent” (iii) “meniscal transplantation” AND “pediatric”, (iv) “meniscectomy” AND “pediatric”, (v) “meniscal transplantation” AND “skeletally immature patient”. No specific fields were designated in the search engines for the keywords [14]. Research on “similar articles” was conducted for the full-text studies selected. Reference lists of potentially relevant original studies were hand-searched to identify any studies not captured using the initial search terms. 

The identified records were screened for duplicates and publication year using a customized spreadsheet. Then, their titles and abstracts were screened for eligibility based on the inclusion and exclusion criteria described above. The full texts of the remaining studies were read to identify the studies for inclusion in the review. Two review authors (Linda Boerci and Francesco Buonanotte) independently conducted the eligibility screening and the full text reading of all potentially eligible retrieved articles. In case of a disagreement regarding inclusion, a consensus was reached through consultation with a third review author (Marco Turati). 

### 2.3. Data Extraction

A customized spreadsheet was used to extract relevant study information such as clinical and demographic characteristics, type of surgery, clinical follow-up and number of complications. A review author (Linda Boerci) performed the data extraction, while a second review author (Daniele Piscitelli) independently verified the data entry.

### 2.4. Methodological Study Quality Assessment

The Newcastle–Ottawa Scale (NOS) [15] was used to grade the methodological quality of each study assessed in the present review. The NOS scale uses a systematic approach based on three specific criteria: selection (S, four points); comparability (C, two points) and exposure (E, three points, which are subdivided into nine sub-criteria: (S1) adequate case definition; (S2) representativeness of the cases; (S3) selection of control; (S4) definition of control; (C1) comparability of cases; (C2) controls based on the analysis; (E1) ascertainment of exposure; (E2) same method of ascertainment for cases and controls; (E3) non-response rate. Each criterion was given a response of either ‘‘Yes”, ‘‘No”, or ‘‘cannot tell”. The maximum score for each study was nine points. Two review authors (Linda Boerci and Francesco Buonanotte) scored the scored the risk of bias, independently. Any disagreements were resolved via discussion with a third review author (Daniele Piscitelli).

## 3. Results

### 3.1. Study Selection

**Figure 1:**
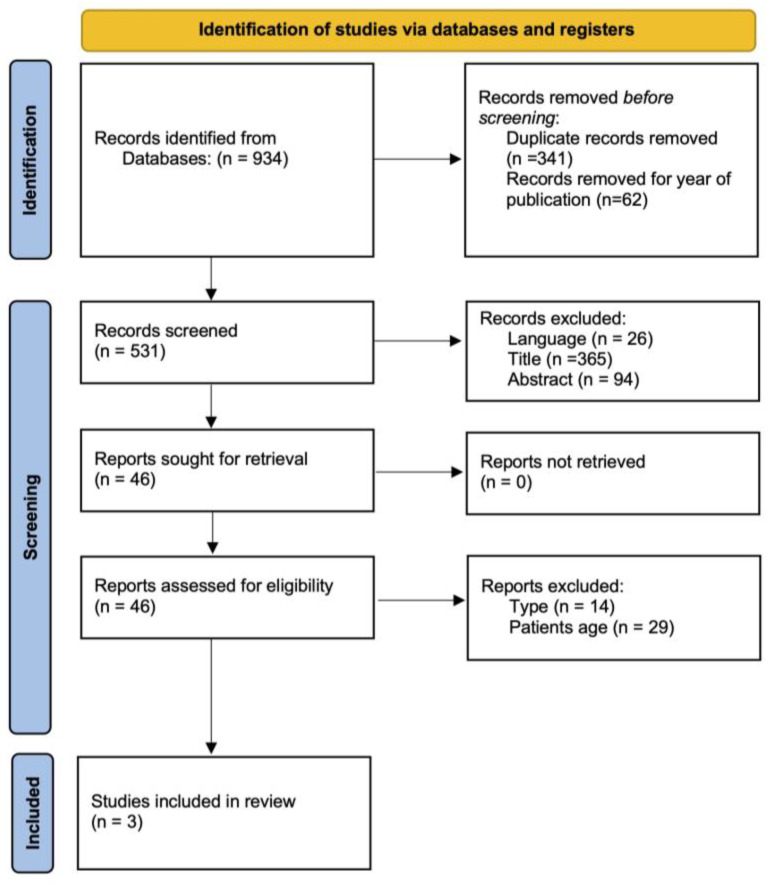
PRISMA flow diagram.

The initial literature search yielded 934 records. After removing duplicate records and applying the publication year range, 531 studies were considered for screening. After excluding studies according to the inclusion criteria, i.e., language (*n* = 26), title (*n* = 365) and abstract (*n* = 84), 46 studies were assessed for inclusion via full-text reading. Fourteen studies were excluded for article type, and twenty-nine were not included due to the study participant age range. In total, three studies [16,17,18] were included and processed for data extraction. Figure 1 depicts the flowchart for study selection. The year of publication ranged from 2016 to 2019.

### 3.2. Study Characteristics

The risk of bias assessment is depicted in Table 1. The study characteristics are summarized in Table 2 and Table 3. We included three retrospective non-controlled case series. All had a population under 18 years of age; two studies [16,17] used a bone-plug technique for the MAT, while a free-graft technique was performed in the third study [18]. The follow-up ranged from 2 to 15 years.

### 3.3. Patient Characteristics

In total, 58 patients (19 males and 39 females) were included. The mean age of this cohort was 15.9 years, and ages ranged from 8 to 18 years of age. All these patients had at least one previous knee surgery: 6 meniscal repairs, 14 meniscectomies of the discoid meniscus, 5 ACL reconstructions, 59 meniscectomies, 1 osteochondral autograft transfer, 1 high tibial osteotomy (HTO), 6 chondroplasties, 3 loose body removals, 1 OCD drilling and 1 microfracture. The most frequently reported indication was post-meniscectomy syndrome. Fourteen patients (24.1%) underwent a previous surgery for discoid meniscus. 

Regarding laterality, the medial side underwent MAT in 17.2% (10 knees) and the lateral meniscus in 82.8% (48 knees). Thirty-five patients (60.3%) were treated with a “bone block” technique, and twenty-three (39.7%) patients were treated with an “all soft tissue graft” technique. Twenty-four patients (41.4%) required associated procedures, including two HTOs, three ACL reconstructions, one ACL revision, eleven autologous chondrocyte implantations (ACIs), three osteochondral allografts and three microfracture procedures.

### 3.4. Outcomes Reported

The mean time to follow-up was 54.3 months. There was no homogeneity in the three studies for collecting pre- and post-surgery scores. The most common patient-reported outcomes reported in the included studies were the International Knee Documentation Committee Questionnaire (IKDC) and its pediatric version (Pedi-IKDC), the Lysholm score, Knee Injury and Osteoarthritis Outcome Score (KOOS), Western Ontario and McMaster Universities Arthritis Index (WOMAC), and the Tegner Activity Scale. The following minimal clinically important differences (MCIDs) were retrieved: Lysholm score (12.3 points), IKDC (9.9 points), KOOS Pain (9.9 points), KOOS Symptoms (9.7 points), KOOS Activities of Daily Living (ADL, 9.5 points), KOOS Sport (13.3), and KOOS Quality of Life (QOL, 14.6) subscales (established in adults, [21]), and the Pedi-IKDC (12.0) (established in children with knee disorders, [22]). The WOMAC reported MCIDs ranging from 13.3 to 36.0 for pain and from 1.8 to 33.0 for function (total knee replacement individuals [23].

Preoperatively, Middleton et al. [18] reported the following scores: IKDC (40.6 ± 12), Lysholm (57.3 ± 18.2), KOOS pain: 70, KOOS symptoms: 61, KOOS ADL 81, KOOS Sports: 43, KOOS Quality of Life (QOL): 28) and Tegner Activity Scale (2, range 0–6). The follow-up occurred at 1, 2, 3, 5 and 7 years, showing an improvement in all scores (IKDC 86.2 ± 3.2; Lysholm 94.5 ± 2.1; KOOS pain 95, KOOS symptoms 90, KOOS ADL 95, KOOS Sports 90, KOOS QOL 70; Tegner Activity Scale 7, range 5–9) at the final follow-up (i.e., seven years). The pre-op scores of Riboh et al. [16] were: KOOS (KOOS pain 64.19 ± 23.20, KOOS symptoms 59.73 ± 17.83, KOOS ADL 75.38 ± 22.35, KOOS sports 35.19 ± 22.89, KOOS QOL 26.62 ± 16.96), WOMAC (pain 5.2; stiffness 3.10; function 16.74) and SF-12 (physical 38.56; mental 54.00), VAS 5, IKDC 40.19 ± 18.98, and Lysholm 43.80 ± 20.37. The patients were evaluated at six months, one year, two years and at a minimum of two years for the final follow-up (ranging from 2–15 years) after surgery, and they reported an improvement of all scores except the IKDC subjective score. At the minimum two-year final follow-up, Kocher et al. [17] reported the following scores: Pedi-IKDC (68.3 ± 4), Lysholm score (55.7 ± 22.3) and Tegner Activity Scale (7). Two out of three patients were able to return to sports at the same level as before the meniscal transplant within nine months postoperatively. 

### 3.5. Complications Reported

Postoperative complications were reported in 16 patients (27.6% of total cases). Eight surgical meniscal procedures were performed (13.7% of total cases): three partial meniscectomies and debridements of the meniscal allograft, one additional partial suture and four removals of suture knots [18].

Seven chondral surgical procedures were reported: five chondroplasties, one removal of a chondral loose body with microfracture of the lateral femoral condyle, and one autologous chondrocyte implantation (ACI) biopsy [16,18]. 

Other secondary surgical procedures reported were: two lysis of adhesions and an arthroscopic control. Middleton et al. [18] reported eight secondary surgical procedures performed after the MAT: one partial meniscectomy, one re-suture of a partial tear, one chondroplasty, four removals of suture knots and one arthroscopy.

Riboh et al. [16] reported a reoperation rate of 22%, with a meniscal reoperation rate of 6%. A total of seven patients underwent new surgery: one arthroscopic lysis of adhesion, one realignment distal femoral osteotomy, and an ACL reconstruction with hamstring, one removal of a chondral loose body with microfracture of the lateral femoral condyle and four chondroplasties with or without partial meniscectomy. No revision was required. The authors did not comment the effects of MAT on limb length or angular alignment.

Kocher et al. [17] reported no growth deformities during the regular postoperative radiological and clinical assessments; on the other hand, one patient required lysis of adhesions along the lateral mini arthrotomy and mobilization under anesthesia. No patients developed a superficial or deep infection. No lower limb discrepancy, growth plate deformities or arrest were reported in all studies.

## 4. Discussion

The purpose of this study was to perform a systematic review of MATs in skeletally immature patients (i.e., children and adolescents) to determine the indications for the procedure and assess the postoperative outcomes in terms of knee pain, function and need for reoperation. Methodologically, the included studies showed a low risk of bias, ranging from six to seven points on the NOS. The indications for meniscus transplantation in skeletally immature patients are the same as in the adult population [17]. Previous surgery for a discoid meniscus (DM) was present in 24% of the patients. Partial meniscectomy is the principal treatment method for symptomatic discoid medial meniscus and can yield promising short-term results. However, because the risk retearing the remaining meniscus is still high in active young patients, when activity modification fails, meniscal allograft transplantation may be an alternative option in complicated DM to diminish pain, improve knee function and prevent or delay cartilage degeneration [24].

Clinically, an important finding was that 41.4% of the patients included in this review required concomitant procedures, the majority of which were related to cartilage damage (31% of cases). Notably, the clinical outcomes and the survival of MAT in isolation and MAT combined with cartilage procedures were similar. The fact that a large portion of these young patients with previous knee surgery had chondral defects or tears underlines the importance of avoiding meniscal deficiency. Nine patients (15.5%) had a previous or an associated ACL reconstruction. This condition pointed out another common indication for MAT in our total population of adolescent patients: irreparable meniscus damage in association with ACL injury [17].

The higher incidence of lateral meniscus transplants in this study population is not only founded in the higher susceptibility of the lateral compartment due to the more significant load in the lateral compartment but also the higher incidence of lateral meniscus loss after a DM corrective procedure [17]. A DM is a type of congenital abnormality in the knee joint that is characterized by a different shape than the typical C-shaped meniscus. A DM is usually circular in shape, and it has an enlarged central portion. Various shapes and levels of instability have been documented in DM. Lateral DM is the most common type of knee anatomical variation [25], but it is challenging to determine its true incidence due to a large number of individuals who have this condition but do not experience any symptoms [26]. The exact cause of DM is still a matter of debate and has not yet been definitively determined. In 1948, Smillie proposed that DM occurs due to a failure in the resorption of the center of the cartilage plate during fetal development [27]. However, a recent systematic review of the embryological data associated with DM’s pathology revealed that the discoid shape was not present in the majority of fetuses, suggesting that it is not a normal stage of fetal development [28].

For patients who are experiencing symptoms such as blockages, pain, decreased quality of life and limitation to participate in sports, surgical treatment is recommended for their specific knee problem [29]. The surgical plan for a symptomatic DM should take into account the menisco-capsular peripheral stability, as classified by Ahn et al. [30], as well as any other potential meniscal tears and concurrent injuries. According to the observations of Deie and his colleagues [31], a complete DM, as classified by Watanabe, is associated with central osteochondritis dissecans, while an incomplete DM is associated with peripheral osteochondritis dissecans.

Different techniques and types of suture were recently described to treat DM surgically; however, Logan et al. [32] recently reported that 33% of pediatric patients with surgically treated lateral DM either underwent reoperation or had ongoing symptoms of the knee at a final follow-up. In this context, meniscal retear is the most common complication (94%) prompting repeat [32]. Considering this data, meniscal transplantation should be evaluated in the case of a loss of meniscal tissue, which is a cause of repetitive meniscal tear and loss of function. Interestingly Yoon et al. [4] conducted a retrospective study to compare the clinical results of MATs after total meniscectomy in a torn discoid lateral meniscus and a non-discoid lateral meniscus. It has been shown that there are no clinical outcome differences for lateral meniscus transplantation after discoid over-resection or non-DM pathology [4].

The cause of most of the subsequent procedures after MAT were related to chondral disease. This emphasizes the risk of early osteoarthritis of the knee and the need for further study on regenerative medicine to delay chondral degradation.

Additional parameters should be considered to better evaluate the impact of MAT in pediatric applications. Meniscal allograft sterilization and preservation should be considered to understand better and evaluate the procedure outcomes. The different types of allografts include fresh, deep-frozen and cryopreserved allografts [7]. However, the cryopreserved allograft remains the most-used preservation technique to reduce procedure costs, improve construct stability and avoid allograft pathogen contamination and tissue-related infection [33].

The allograft composition should also be evaluated. Further studies are needed to characterize healthy constructs that are able to induce tissue regeneration. The extracellular matrix (ECM) plays a key role in maintaining the physiological condition of tissues, including cell fate regulation and, consequently, tissue repair [34]. Collagens, glycoproteins, and proteoglycans mainly constitute the meniscal ECM. Even if the total proteome is not fully characterized, it is well known that alterations to the ECM composition, stiffness, and architecture induce pathological phenomena, including osteoarthritis [35]. Recently, proteomic analysis has been used on osteoarthritic medial meniscal tissues to characterize a proteome profile in radial zones, showing differentially expressed ECM proteins in lateral and medial lesions [36]. A global analysis taking into consideration ECM signatures, architecture and physical features associated with a patient-personalized approach that includes age and the size of defects size could improve the design of new clinical strategies in skeletally immature patients with meniscus-deficient knees.

The clinical outcomes that have been reported in the three included studies are psychometrically sound. Riboh et al. (2016) [16] and Middleton et al. (2019) [18] reported the KOOS and its subscale, while the IKDC and Lysholm questionaries were shown in all studies. The KOOS, IKDC and Lysholm questionaries are clinically relevant outcome measures. To study and refine their measurement properties, the KOOS and IKDC have been investigated via Rasch Analysis. Conflicting findings were reported (see [37,38,39]). This is not surprising as Rasch Analysis is a strict model for testing the measurement requirements of patient-oriented outcome [40,41]. Notably, the KOOS has been recommended as one of the patient-reported outcome measures to be administrated when evaluating endpoints or follow-up in clinical trials [42]. However, according to a recent study by van der Velden et al. [43], in children with knee disorders and injuries, the Pedi-IKDC should be preferred for use in clinical practice for its administration time and acceptability. With respect to clinically meaningful changes defined by MCIDs, the values retrieved (see Section 3.4., Outcomes Reported) were not studied in skeletally immature individuals undergoing MAT. Notably, the Pedi-IKDC used in the study by Kocher et al. (2016) [17] had an MCID reported in children with various knee disorders, but the study authors did not administrate it as a pre-operative score; thus, no changes were reported. It should be noted that the MCID is intimately linked with the demographic and baseline clinical characteristics and the treatment used for reporting the responsiveness of the outcomes [44,45,46]. Therefore, further psychometric studies should investigate the MCID in children and adolescents with meniscal lesions.

The present study has some limitations that must be acknowledged. First, our sample size is limited because we selected only studies with patients under 18 years of age. Second, the original studies that were analyzed were not specifically designed to evaluate the impact of MAT on the length or alignment of the limbs. The findings of the present systematic review rely on two prospective studies and one retrospective study, resulting in low-quality evidence. In addition, 41.4% of the patients required associated procedures that may have confounded the results [16]. This calls for caution when interpreting the findings. Despite these limitations, this systematic review offers a comprehensive and updated overview of the available literature on MAT in the pediatric population, which can serve as a starting point for future research. High-quality studies on MAT in skeletally immature patients are needed to provide evidence regarding the clinical effectiveness of the procedure and its impact on health outcomes in later adulthood. Further studies should investigate the outcomes in medial meniscus MAT.

## 5. Conclusions

In conclusion, we have observed that the most frequently reported indications were post-meniscectomy syndrome and DM (24.1%). Good clinical outcomes are reported; however, long-term results are still missing, and no specific studies on degenerative knee osteoarthritis are present. In a pediatric population, MAT remains a challenging procedure with a high rate (27.6%) of postoperative complications; however, no lower limb discrepancy or growth plate deformities were reported. Further observations are required to evaluate the long-term success of this procedure. 

## Figures and Tables

**Table 1:** The Newcastle-Ottawa Scale (NOS).

Study	Sample Selection				Comparability		Outcome			Total
	Adequate Case Definition	Representativeness of the Cases	Selection of Control	Definition of Control	Comparability of Cases	Controls Based on the Analysis	Ascertainment of Exposure	Same Method of Ascertainment for Cases and Controls	Non-Response Rate	
Riboh et al. (2016) [16]	★	★			★	★	★	★	★	7
Kocher et al. (2016) [17]	★	★			★	★	★	★		6
Middleton et al. (2019) [18]	★	★			★	★	★	★	★	7

Star (★) = item present.

**Table 2:** Study characteristics.

Study	Subjects	Surgical Technique	Lateral vs. Medial Meniscus	Associated Procedures
Riboh et al. (2016) [16]	*n* = 32 (23 F; 9 M) Mean age 15.4 years (13–16 years)	Bone-plug technique (bridge-in-slot technique [19])	5 medial menisci 27 lateral menisci	*n* = 18, 11 ACI; 2 ACL reconstructions; 1 OATS; 3 Osteochondral allografts; 1 HTO
Kocher et al. (2016) [17]	*n* = 3 (2 F; 1 M) Mean age 12.6 years (9–14 years)	Physeal-sparing bone-plug technique [17]	1 medial meniscus 2 lateral menisci	-
Middleton et al. (2019) [18]	*n* = 23 (14 F; 9 M) Mean age 17 years (8–18 years)	Free-graft technique with sutures through bone tunnels tied over a bone bridge on the anteromedial tibia [20]	4 medial menisci 19 lateral menisci	*n* = 6, 1 HTO; 1 ACL reconstruction; 1 ACL revision; 3 microfracture procedures

ACI, autologous chondrocyte implantation; ACL, anterior cruciate ligament; OATS, osteochondral autograft transfer System; HTO, high tibial osteotomy.

**Table 3:** Summary of reported outcomes in children and adolescents treated with MAT.

Study	Follow-Up	Pre-Operative Scores	Final Follow-Up	Subsequent Procedure
Riboh et al. (2016) [16]	Mean: 7.2 ± 3.2 years (range 2–15 years)	IKDC: 40.19 ± 18.98	IKDC: 65.02 ± 17.70	7 reinterventions: 1 femoral osteotomy; 3 chondroplasty; 2 meniscectomy; 1 lysis of adhesions; 1 removal of loose body + ACLR
		Lysholm: 43.80 ± 20.37	Lysholm: 58.52 ± 17.92	
		KOOS pain: 64.19 ± 23.20 KOOS symptoms: 59.73 ± 17.83 KOOS ADL: 75.38 ± 22.35 KOOS sports: 35.19 ± 22.89 KOOS QOL: 26.62 ± 16.96	KOOS pain: 76.57 KOOS symptoms: 72.36 KOOS ADL: 90.09 KOOS sports: 62.61 KOOS QOL: 54.89	
Kocher et al. (2016) [17]	Mean: 31± 20 months	Pedi-IKDC: Not Reported	Pedi-IKDC: 68.3 ± 4	1 lysis of adhesion
		Lysholm: Not Reported	Lysholm: 55.7 ± 22.3	
		Tegner: Not Reported	Tegner: 7	
Middleton et al. (2019) [18]	Median: 3.8 years (range 0.2–7.8 years)	IKDC: 40.6 ± 12	IKDC: 86.2± 3.2	8 reinterventions; 1 partial meniscectomy; 1 re-suture of partial tear; 1 chondroplasty PFJ; 1 arthroscopy; 4 removals of suture knots
		Lysholm: 57.3 ± 18.2	Lysholm 94.5 ± 2.1	
		KOOS pain: 70 KOOS symptoms: 61 KOOS ADL: 81 KOOS sports: 43 KOOS QOL: 28	KOOS pain: 85 KOOS symptoms: 78 KOOS ADL: 88 KOOS Sports: 69 KOOS QOL: 65	

Abbreviations: IKDC, International Knee Documentation Committee Questionnaire; KOOS, Knee Injury and Osteoarthritis Outcome Score; ADL, activities of daily living; ACLR, Anterior Cruciate Ligament Reconstruction; QOL, Quality of life; PFJ patella–femoral joint.

## Data Availability

The data that support the findings of the current study are available from the corresponding author (Daniele Piscitelli) upon reasonable request.
